# The interplay of grandparental investment according to the survival status of other grandparent types

**DOI:** 10.1038/s41598-022-18693-9

**Published:** 2022-08-23

**Authors:** Samuli Helle, Antti O. Tanskanen, Jenni E. Pettay, Mirkka Danielsbacka

**Affiliations:** Department of Social Research, University of Turku, Turku, Finland

**Keywords:** Developmental biology, Evolution

## Abstract

Inclusive fitness theory predicts that grandparental investment in grandchildren aims to maximise their inclusive fitness. Owing to an increasing overlap between successive generations in modern affluent populations, the importance of grandparental investment remains high. Despite the growing literature, there is limited knowledge regarding how the survival status of different grandparent types influences each other’s investment in grandchildren. This question was studied by using the Involved Grandparenting and Child Well-Being Survey, which provided nationally representative data of English and Welsh adolescents aged 11–16-years. We applied Bayesian structural equation modeling (BSEM) where grandparental investment in grandchildren was modelled using multi-indicator unobserved latent variable. Our results showed that maternal grandmothers’ investment was increased by having a living maternal grandfather but not vice versa. Having a living maternal grandmother was also associated with decreased investment of paternal grandparents while the opposite was not found. These findings indicate that the association between the survival status of other grandparents and the focal grandparents’ investment varies between grandparent types.

## Introduction

Based on inclusive fitness theory, grandparents can increase their inclusive fitness by investing in their grandchildren with whom they share, on average, 25% of the same genes^1^. In historical and traditional populations, the presence of post-reproductive grandmothers has been shown to be related to increased fertility of adult children and improved early-life survival of grandchildren^2–7^. From an inclusive fitness perspective, grandparental investment can be defined as actions of grandparents that have the potential to improve the fitness of grandchildren at the expense of any direct fitness costs for the grandparents themselves^8^. Owing to declining fertility rates in several contemporary high-income post-industrial countries, grandparents currently might have fewer grandchildren than before, meaning that grandparents can potentially invest more in grandchildren overall because of fewer alternative investment options^9^. Although grandparents have been found to increase their investment in grandchildren when ceasing their own fecundity^10^, they may stop some forms of investment that become too costly as they grow very old (e.g. survival-enhancing care in historical and traditional populations^11^), whereas emotional support (e.g. advice and listening) to grandchildren may continue until grandparents reach a very old age^12^.

A wide range of studies have shown biased grandparental investment in contemporary Western societies according to grandparental sex and lineage; this bias spans various substantive forms of investment, including contact frequency, emotional closeness, financial support and physical care^13,14^. One of the most frequent findings in grandparental investment research is that maternal grandparents invest more than paternal grandparents and, among maternal grandparents, it is the grandmothers that invest the most^15–19^. Moreover, prior studies have shown that the investment of maternal grandmothers is least facultative compared to other grandparent types. For example, maternal grandmothers seem to invest the most in grandchildren regardless increased geographical distance^20^ or decreased reproductive value of grandchildren^21^. Like in sexually reproducing species where the investment of males in offspring is tied to their paternity uncertainty^8^, theoretical background for these findings stem from paternity confidence that favors the investment by maternal grandmothers on their daughter’s progeny. Only maternal grandmothers can be fully certain of their relatedness to grandchildren while other grandparent types cannot^19^. Also, a more general bias in investment towards matrilateral kin occurs, due to differential fitness-returns through female versus male relatives^15,16^.

Despite the vast amount of research on biased grandparental investment, only a handful of studies so far investigate whether the death of one grandparent has any effect on other grandparents’ levels of investment. Some studies have found that there tends to be a decrease in grandfathers’ investments in their grandchildren when their spouses (i.e., grandmothers) are deceased^22–24^, although other studies find that this effect is either very small or negligible^18,25^. The death of grandfathers tends to have no impact on the investment of grandmothers^24^. These prior findings thus indicate that when grandmothers are investing in their grandchildren, their spouses (i.e., grandfathers) will be “incidentally exposed” to the grandchildren as well, which strengthens grandfathers’ investment in grandchildren^9^. Within a lineage, the presence of a living spouse can be assumed to boost the investment of the focal grandparent owing to, for example, a more secure financial situation, as suggested in some prior studies^22,23^.

Perhaps the most important limitation of these prior studies is that they investigate the influence of grandparental survival status only within a lineage. Because all dyadic relationships between grandparents and grandchildren tend to be linked to one another^26^, the survival status of a grandparent(s) also could have an impact on the investment of grandparents on the other side of the family. When the grandparents from another lineage are deceased, the investment of a focal grandparental lineage could be increased because those grandparents are compensating for the lack of resources from the deceased lineage. In support of this possibility, prior studies indicate that high investment of maternal grandparents in grandchildren tends to diminish the investment of paternal grandparents^27,28^.

In most studies on grandparental investment, only one or a few observed variables measuring grandparental investment have been examined at a time and those variables have varied between studies^9,14^. This approach has shortcomings if the variables included in the study fail to reliably represent the realm(s) of grandparental investment: some constructs such as investment in progeny may be hard to reliably measure, on both theoretical and practical grounds, using just a few variables^29^. The resulting inaccuracy between the recorded variables and the scientific construct of interest (i.e. grandparental investment in the current study) results in measurement error in the construct and may seriously bias, for example, inferences concerning the relationship between grandparental investment and grandchild outcomes, thus reducing the generalizability of such results^30^. Moreover, when one is interested in factors influencing grandparental investment (e.g., grandparental sex and lineage), using poor proxies of the true investment underestimates the amount of variance explained in investment and reduces the statistical power needed to find patterns in the data^30^. Therefore, the field could benefit from using latent unobserved variables to obtain more reliable inferences.

To the best of our knowledge, no prior study has examined whether the survival status of other grandparents is associated with focal grandparents’ investment in their grandchildren, simultaneously, both within and between lineages. The present study fills this gap by using nationally representative data from England and Wales, where adolescent grandchildren provide information on grandparental investment. Therefore, these data may provide the most reliable assessment of true grandparental investment by grandparent type^9^. To maximize statistical power, we also used multiple grandparental investment variables to model one latent construct that measured grandparental investment in grandchildren. Based on paternity uncertainty and prior findings showing that maternal grandmothers invest the most in grandchildren, we predict that the investment of maternal grandmothers is least likely to be affected by the survival status of other grandparents (“independency effect”)^20,21^. In the case of within-lineage effects, we may either expect that grandfathers are incidentally exposed to grandmothers thus the death of grandmothers diminishes the investment of grandfathers (“incidental exposure effect”)^9^ or that having a spouse alive may increase the investment of the focal grandparent (either female or male) as the result of increased household resources (“spouse effect”)^22,23^. Considering the between-lineage effects, grandparents from one lineage may compensate for the lack of investment from another lineage (e.g. if the grandparents from that lineage are deceased) and hence maternal grandparents may be assumed to invest more if paternal grandparents are deceased, and vice versa (“compensatory effect”)^27,28^. In the case of between-lineage effects, the other possibility is that the death of maternal grandmothers will increase the investment of paternal grandparents because when maternal grandmothers (i.e., the key investors) are deceased there is more need and room for the investment of paternal grandparents (“absence of maternal grandmother effect”)^27,28^.

## Methods

### Data

We used the Involved Grandparenting and Child Well-Being 2007 survey, recruited by GfK National Opinion Polls, which is a nationally representative sample of English and Welsh adolescents aged 11–16^31–33^. The sample resulted from the distribution of surveys to schools. From the 103 randomly selected schools, in which classes were randomly chosen for survey distribution, 70 schools returned the questionnaires (response rate: 68%). Respondents completed the questionnaire in a school classroom, and the original sample included 1566 adolescent^31^. 89% of the respondents identified as white ethnicity, 3.5% identified as black or afro-Caribbean, and 4.3% identified as Asian or mixed ethnicity (for more details on respondents, please see e.g.^18^). When filling in the questionnaire on grandparental investment, respondents were asked to answer questions for only those grandparents who were still alive. Hence, only those respondents who had at least one living grandparent (n = 1488) were considered in the analyses. We also excluded those children from the analyses (n = 58) who were co-residing with their grandparents; such cases are unusual in England and Wales, and it is difficult to estimate the level of grandparental investment in three-generational compared to two-generational households. Nineteen grandchildren were further dropped from the analysis because they had no response in any of the questions asked or had missing data in their age (mean = 13.39, s.d. = 1.41). The total number of children included in the analyses was hence 1411. The proportion of living maternal grandmother, maternal grandfather, paternal grandmother, and paternal grandfather were 83.7%, 68.8%, 73.2%, and 57.1%, respectively.

To measure grandparental investment in their grandchildren, we used questions developed by Elder and Conger^34^. From the list of all questions available, we chose four questions that directly measured grandparental investment; these were: “how often do you see them” (Q15), “their grandparents had looked after them” (Q26), “they could depend on their grandparents” (Q27), and “provided financial assistance or help” (Q38). Question Q26 was reverse-scaled to match the meaning and ordering of the other scales. Questions Q15, Q26, and Q27 were measured on a 4-point Likert-type item ranging from 1 = *not at all/never* to 4 = *a lot/every day*, and Q38 was measured on a 3-point Likert-type scale ranging from 1 = *never* to 3 = *usually.*

### Statistical analysis

**Figure 1:**
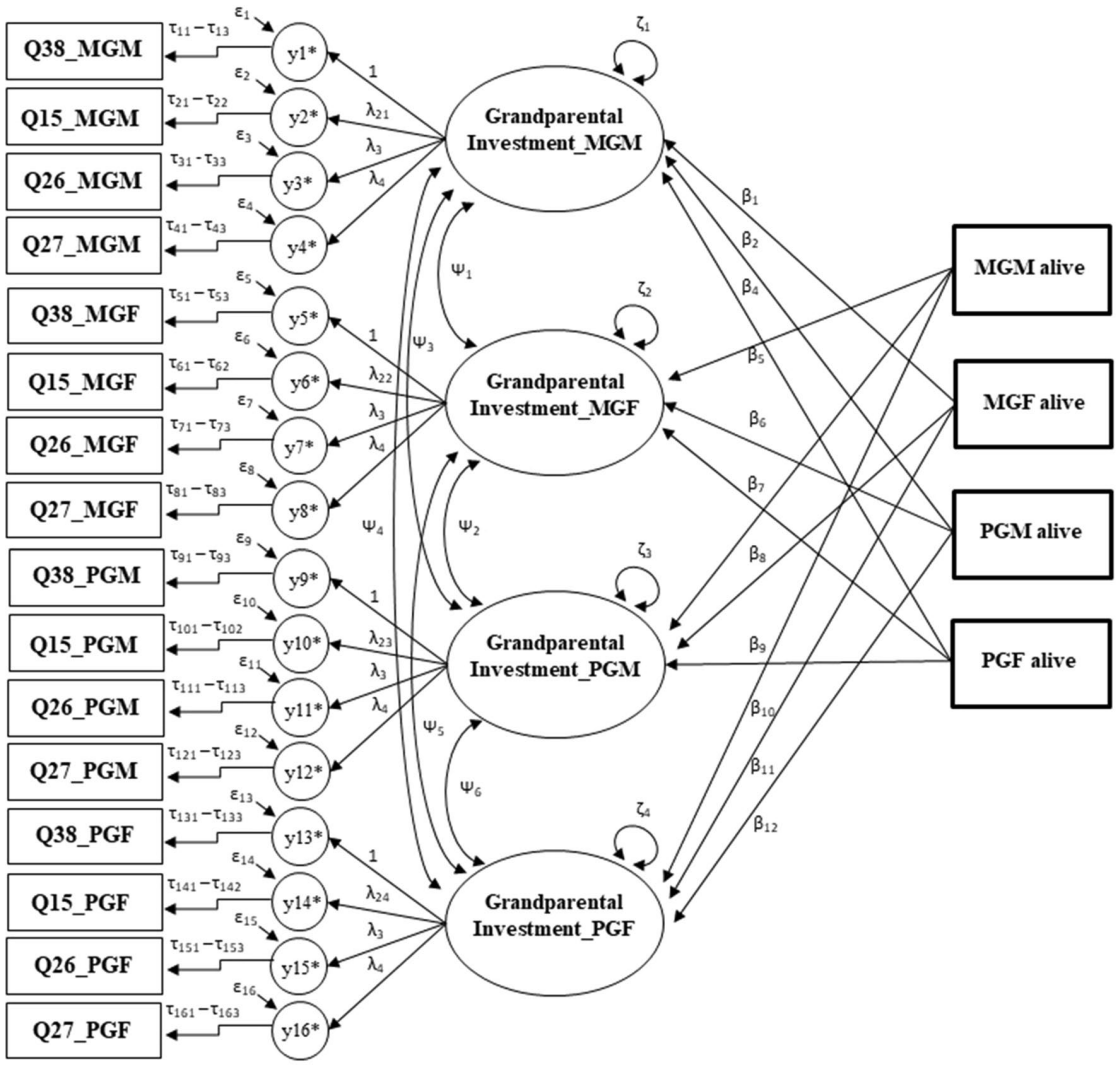
A graphical representation of the structural equation model used to examine how the survival status of other grandparents influenced a focal grandparent’s investment in their grandchild. Observed variables are represented as boxes (please note that the covariate grandchild age is omitted here for simplicity) and unobserved latent variables as circles. Single-headed straight arrows have three functions here: (i) when pointing from a latent variable to another latent variables (i.e., underlying normally-distributed latent variable (e.g., y1*–y4*) for each observed question per grandparent (Q15: “how often do you see them”, Q26: “their grandparents had looked after them”, Q27: “they could depend on their grandparents” and Q38 “provided financial assistance”. The suffixes _MGM, _MGF, _PGM and _PGF denote maternal grandmother, maternal grandfather, paternal grandmother, and paternal grandfather, respectively), they represent reflective linear loadings (λ’s) of the latent; (ii) when pointing at those underlying latent variables of the questions asked from grandchildren, they represent their unique residual errors (ε’s); and (iii) when pointing from observed independent variables to latent grandparental investments, they represent structural path coefficients (β’s). Single-headed arrows with a step denote a non-linear association, modeled as a probit link function by thresholds (e.g., τ_11_–τ_13_, τ_21_–τ_22_ etc.), linking the latent variables and their indicators together. Double-headed arrows represents the error variances of the latent “grandparental investment” variable (ζ’s) and their covariances (Ψ’s).

We used Bayesian structural equation modelling (BSEM) with multiple-indicator latent variables^35^ to simultaneously examine how the survival status (dead or alive) of other grandparents influenced subjects’ investment in their grandchild (Fig. 1). The response variable was an unobserved latent variable representing the construct “grandparental investment” in their grandchild, measured by four effect indicators (i.e., the latent variable was assumed to cause variation in its indicators) that were the questions asked from the grandchildren about their grandparents’ involvement in their life (i.e., Q15, Q26, Q27, and Q38)^35^. This means that each question contributed differently to how much each grandparent invested in their grandchild. In other words, we did not aim to model each specific component of grandparental investment separately but considered grandparental investment in their grandchildren as an unmeasured (i.e., measured with measurement error) construct including all its components. The question “provided financial assistance or help” was regarded as the most relevant and reliable observed indicator variable of grandparental investment and was thus used as a marker indicator of the latent variable by fixing its unstandardized loading to unity (Fig. 1). All indicators of the latent variable were treated as ordinal with a probit link function. Therefore, the loadings connecting the latent variable to its indicators can be interpreted as the extent to which a one-unit increase in the latent variable score changes the predicted probit index (z-score) in standard deviation units. In SEM with categorical latent variable indicators with probit link, it is assumed that the categories of observed ordinal variables are determined by the thresholds (the number of categories in the observed variable minus one) in the underlying normally distributed latent variable^35^. These latent variables then become the indicators of the main latent variable (here, grandparent’s investment), which are, in turn, associated with the ordinal observed variables by the respective threshold structure (Fig. 1)^35^. Note that when the latent variable with discrete indicators is regressed on independent variables, these coefficients are linear regression coefficients. To be able to compare grandparental investment among the grandparent types using latent variables, a certain level of measurement invariance needs to be established between the grandparent types^36^. For these data, we relied on partial measurement invariance, where one of the four factor loadings was non-invariant between the groups (thresholds were found to be invariant among grandparent types)^21^. Moreover, as commonly done in dyadic analyses^37^, we allowed for covariances among the errors (i.e., unexplained parts of the variation) of the latent variables to account for unmeasured factors that influenced grandparental investment within lineages (Fig. 1). Between-lineage error covariances were also modelled because the same grandchild evaluated investment for each grandparent, which may also have induced correlations between the responses (Fig. 1).

We did not have access to direct measures of grandparental wealth or other resources for these data, which are likely important within-lineage confounders of the effect of grandparent’s survival status on his or her spouse’s investment in grandchildren^17^. The data set used here did include variables like marriage and employment status of grandparents at the time of the research, which could be regarded, to a varying degree, as proxies of within-lineage resources available for grandparents (e.g., money and time). Another likely confounder of the survival status-investment-relationship is grandparental age, as a focal grandparent’s age is likely linked to the survival status of his or her partner (i.e., older grandparents, particularly grandmothers owing to the general survival advantage of women over men, are more likely to be widows) and his or hers investment in grandchildren (i.e., very old grandparents are likely unable to invest much). However, a major drawback of these data is that information on grandparental age, marriage status, and employment status (along with the data on investment in grandchildren) were only recorded for those grandparents who were alive during the study. Including those potential confounders into the model using the default listwise procedure therefore would have invalidated the whole analysis since all the deceased grandparents would have been omitted from the analysis. As grandparent’s survival status can also be regarded as missing data indicators, handling missingness in grandparent-specific covariates by bringing them into the model by estimating their means, variances, and covariance^38^, or using multiple imputation would have resulted in non-estimable parameters (i.e., covariances among covariates and survival status variables)^39^. Consequently, we were unable to include grandparent-specific independent variables in the analysis. Only grandchild age was included as a covariate to improve the efficiency of the regression estimates^21^. The correlation matrix of the variables used in the analysis can be found in the supplementary materials (Tables S1 and S2).

Instead, we performed a robustness check on our base model to evaluate the impact of unobserved confounding (i.e., shared causes for both independent and dependent variables or omitted variable bias) on the association between grandparental investment and the survival status of other grandparent types, as recently described in Harring et al.^40^ (please also see Imai et al.^41^). In this method, the effect of a potential unmeasured confounder, or confounders, is mimicked by a phantom variable that affects both the predictor (i.e., survival status of other grandparents) and the outcome (i.e., investment of a focal grandparent). Phantom variables are latent variables without any indicators, precluding the need for actual data. Instead, the mean and variance of the phantom variables are fixed constants, usually set to zero and unity, respectively^40^. The rationale is to examine the sensitivity of the original conclusions when one adds the phantom variable as an unmeasured confounder(s) into the model and varies the strength of the expected confounding^40^.

As discussed above, one potential confounder of the within-lineage effect between the focal grandparent’s investment and the survival status of his or her spouse is the socioeconomic status or resource availability of the grandparents. It is likely that high socioeconomic status in grandparents improves both their survival (e.g., via healthier lifestyle) and increases their ability to invest in their grandchildren. On the other hand, grandparental age is likely a within-lineage confounder that has negative effects both on grandparents’ survival and their investment in grandchildren. However, since the consequences of these two confounders (i.e., resource availability having positive and grandparental age having negative effects on both the outcome and the independent variable) on the association of main interest here are quantitatively the same^42^, we concentrated only on the case of confounding by resource availability in our sensitivity analysis. While the signs of the suspected confounders are usually easy to argue, the strength of these effects is usually arbitrary without strong prior subject knowledge. Two scenarios were evaluated here. First, the level of confounding was assumed to be roughly equal to the maximum within-lineage effect size observed between the survival status of a spouse and focal grandparent’s investment. Second, we doubled this level of confounding.

We applied Bayesian inference using the Gibbs sampler for the Markov Chain Monte Carlo (MCMC) algorithm to draw posterior distribution to our model parameters. The median of posterior distribution was used as a point estimate and the highest posterior density (HPD) was used for 95% (credibility) interval estimation. Missing data in indicators (which were treated as response variables) were assumed to be missing at random and handled by Bayes as a full-information estimator. Non-informative normally distributed priors were used for structural regression coefficients (hyperparameters for prior mean and variances = N(0, 100^2^)), factor loadings (N(0, 5)), thresholds (N(0, 1)), and non-informative inverse Wishart priors for error variances (IW(1, 5)), and covariances (IW(0, 5)) of latent variables.

Three chains with a total of 300,000 iterations were run, thinned by every 50th iteration due to some strong autocorrelation among the threshold parameters, with a burn-in of 150,000 iterations. The convergence of MCMC chains was determined using a potential scale reduction factor that compared the estimated between-chains and within-chains variances for each parameter^43^. In general, values below 1.2 and 1.1 are considered to indicate good convergence of the chains. The potential scale reduction factor for our model was 1.002 after the iterations used here, suggesting appropriate convergence. We also inspected the individual trace plots of individual parameters as well as their autocorrelation plots, confirming convergence. Mplus 8.7 was used for all data analyses^44^.

### Ethical approval and consent to participate

This study was approved, and the research was performed in accordance with the guidelines of University of Oxford Research Committee. All the participants and their parents gave a written consent to participate in the study in accordance with the Declaration of Helsinki.

## Results

### The base model

The results of the base model showed that maternal grandmothers’ investment in grandchildren was increased by having a living paternal grandmother (Table 1; for complete model results, please see supplementary material Table S3). In addition, both paternal grandparents’ investments were negatively influenced by having a living maternal grandmother (Table 1). In terms of marginal effects, a grandchild’s conditional probability of scoring “never” and “usually” for the question setting scale of the latent variable “grandparental investment” (i.e., “provided financial assistance or help?”) when the paternal grandmother was dead were 8.1% and 48.2%, respectively. The corresponding probabilities were 5.9% and 54.5% for grandchildren whose paternal grandmother was alive. When the maternal grandmother was alive, a grandchild’s conditional probabilities of scoring “never” and “usually” for the marker question for the paternal grandmother were 10.1% and 43.8%, respectively. The corresponding probabilities were 6.9% and 52.1% for grandchildren whose maternal grandmother was deceased. Similarly, a living maternal grandmother resulted in a grandchild’s conditional probability of scoring “never” and “usually” for paternal grandfather being 13.8% and 39.5%, respectively. If the maternal grandmother was dead, these conditional probabilities were 9.7% and 47.6%.

**Table 1:** Structural regression coefficients of a Bayesian structural equation model on how the survival status of maternal grandmothers (MGM alive; dead as a reference class) and grandfathers (MGF alive) as well as paternal grandmothers (PGM alive) and grandfathers (PGF alive) influenced each other’s investment in grandchildren.

	Median	95% C.I	One-tailed p-value	
**Maternal grandmother**				
MGF alive	0.157	0.060, 0.256	0.001	
PGM alive	0.057	−0.047, 0.155	0.134	
PGF alive	0.059	−0.030, 0.150	0.101	
**Maternal grandfather**				
MGM alive	−0.044	−0.199, 0.104	0.291	
PGM alive	−0.033	−0.160, 0.088	0.297	
PGF alive	0.009	−0.101, 0.114	0.437	
**Paternal grandmother**				
MGM alive	−0.207	−0.349, −0.067	0.03	
MGF alive	−0.001	−0.117, 0.119	0.494	
PGF alive	0.058	−0.055, 0.179	0.165	
**Paternal grandfather**				
MGM alive	−0.207	−0.367, −0.050	0.005	
MGF alive	−0.025	−0.159, 0.108	0.360	
PGM alive	0.062	−0.123, 0.239	0.248	

For full results, please see the supplementary material Table S3.

For a positive posterior median, one-tailed p-value gives the proportion of posterior distribution that is below zero, and for a negative posterior median the proportion of posterior distribution that is above zero is given.

*95% C.I.* 95% credibility interval of the posterior median of coefficients.

After taking the survival status of grandparent types and grandchild’s age into account, we observed non-zero positive covariances among all the latent variables presenting grandparental investment (Table S3). Table 2 shows that the highest correlations among grandparental investment were unsurprisingly within-lineages whereas correlations between grandparent’s investment between-lineages were much lower.

**Table 2:** Correlation matrix of latent variables representing grandparental investment in grandchildren.

	Investment_MGM	Investment_MGF	Investment_PGM	Investment_PGF
Investment_MGM	1			
Investment_MGF	**0.881**	1		
Investment_PGM	*0.263*	*0.303*	1	
Investment_PGF	*0.219*	*0.263*	**0.958**	1

Cells filled in with bold and italics denote within- and between-lineage correlations of grandparental investment, respectively. The suffixes _MGM, _MGF, _PGM and _PGF denote maternal grandmother, maternal grandfather, paternal grandmother, and paternal grandfather, respectively.

### Sensitivity analysis

Sensitivity analysis showed that an equal-sized confounding was not enough to change our inferences based on the base model. However, confounding of double the size in magnitude of the corresponding base model effect sizes changed the result of the positive association between maternal grandmother’s investment and her spouse being alive to an association not differing statistically from zero (Table 3). Also, doubling the size of the confounding also resulted in a negative influence of a living maternal grandmother on the maternal grandfather’s investment (Table 3). A living maternal grandmother’s negative influence on the paternal grandparents’ investment remained unchanged because the confounding considered here concerned only within-lineage effects.

**Table 3:** Sensitivity analysis of the regression coefficients and their 95% credibility intervals (C.I.) for other grandparent types’ influence on a focal grandparent’s investment from the base model when there was either 1- or twofold within-lineage confounding. Bolded cases indicate statistically non-zero coefficients.

	No confounding		Onefold confounding		Twofold confounding	
	Median	95% C.I.	Median	95% C.I.	Median	95% C.I.
**Maternal grandmothers**						
MGF alive	**0.157**	**0.060, 0.256**	**0.116**	**0.023, 0.216**	0.003	−0.091, 0.97
PGM alive	0.057	−0.047, 0.155	0.059	−0.043, 0.158	0.062	−0.040, 0.160
PGF alive	0.059	−0.030, 0.150	0.059	−0.031, 0.149	0.060	−0.029, 0.150
**Maternal grandfathers**						
MGM alive	−0.044	−0.199, 0.104	−0.089	−0.222, 0.072	**−0.206**	**−0.350, −0.056**
PGM alive	−0.033	−0.160, 0.088	−0.031	−0.148, 0.099	−0.027	−0.145, 0.100
PGF alive	0.009	−0.101, 0.114	0.010	−0.098, 0.119	0.011	−0.101, 0.116
**Paternal grandmothers**						
MGM alive	**−0.207**	**−0.349, −0.067**	**−0.203**	**−0.340, −0.066**	**−0.202**	**−0.342, −0.066**
MGF alive	−0.001	−0.117, 0.119	0.000	−0.118, 0.123	−0.001	−0.113, 0.129
PGF alive	0.058	−0.055, 0.179	0.054	−0.064, 0.167	0.038	−0.071, 0.162
**Paternal grandfathers**						
MGM alive	**−0.207**	**−0.367, −0.050**	**−0.208**	**−0.368, −0.051**	**−0.206**	**−0.361, −0.049**
MGF alive	−0.025	−0.159, 0.108	−0.025	−0.164, 0.108	−0.026	−0.168, 0.104
PGM alive	0.062	−0.123, 0.239	0.050	−0.140, 0.219	0.033	−0.155, 0.202

## Discussion

The current study presents, to the best of our knowledge, the first investigation of the interplay of grandparental investment according to the survival status of other grandparent types both within- and between-lineages. Our results showed that maternal grandmothers’ investment in their grandchildren was elevated if the maternal grandfather was alive. However, this finding was not fully robust to confounding as this association disappeared statistically if the magnitude of confounding was twice as large as the association evaluated by the sensitivity analysis. Confounding of such a magnitude also resulted in a negative association between a maternal grandmother’s survival and her spouse’s investment. Furthermore, we found a reduced investment of paternal grandparents if the maternal grandmother was alive. The effect size of this association was equal among both paternal grandparents, largely because the correlation of grandparental investment between paternal grandparents was very high (correlation = 0.958). The between-lineage results of grandparental survival status were not directly exposed to sensitivity analysis for confounding because we only modelled within-lineage confounding: we considered it very unlikely that, for example, maternal grandparents’ resource availability would causally affect the survival status and investment in grandchildren of paternal grandparents. Among paternal grandparents, the survival status of a partner was unrelated to investment in grandchildren in both sexes and this conclusion was not changed by the magnitude of confounding examined here.

Previous literature has suggested that if any associations exist between grandparental investment and her/his spouse’s death, they are negative: i.e., they reduce the focal grandparent’s investment^22–26^. Our results partly support this “spouse effect” by indicating that increased maternal grandmother’s investment in grandchildren was associated with having a living maternal grandfather (but not vice versa). No such associations were observed, however, in paternal grandparents. Such an association may arise if maternal grandfathers’ presence indicates that there are more resources in the household, and maternal grandmothers are able to use these extra resources to the advantage of grandchildren by increasing their investment in those grandchildren. That is, the influence of maternal grandfathers’ survival status could be mediated via more resources to be invested by maternal grandmothers. The opposite seems not to hold in these data as the maternal grandmother’s survival status was not related to the maternal grandfather’s investment; only when we introduced confounding twice as strong as the effect sizes observed did the influence of maternal grandmother being alive on her spouse’s investment turn out to be negative—a result that seems counterintuitive. This disagrees with the “incidental exposure effect” suggesting that maternal grandfathers’ investment in grandchildren would be a mere by-product of the their spouse’s investment (i.e., maternal grandmothers)^9^. Furthermore, there was no support detected for the prediction that paternal grandfathers are incidentally exposed to the investment of paternal grandmothers.

In general, prior studies have indicated that women are more likely to invest extra resources to benefit their families than men^9,13^. In turn, the findings related to survival status and investment of maternal and paternal grandmothers show that kin relations may present themselves in different ways according to grandmaternal lineage. According to the present findings, the presence of maternal grandmothers, who typically invest the most of all grandparent types in their grandchildren even in modern patrilineal societies^15^ and in unfavourable family conditions^21^, seemed to reduce the investment of paternal grandparents. Such an association seems to be asymmetric as the survival status of the paternal grandparents was not associated with the investment of maternal grandparents. These findings support the “absence of maternal grandmother effect” and are widely in line with evolutionary theories of grandparental investment. From the perspective of paternity uncertainty in particular, if grandparents have an option to invest either in their daughters’ or sons’ children they should direct their investment primarily towards their daughters’ children^27^. In turn, when the maternal grandmother is deceased, paternal grandparents may compensate for this loss by investing more in their grandchildren.

The data used in the present study have several strengths. According to the data, the adolescents were the respondents providing information on grandparental investment and background variables related to themselves and grandparents. Grandparents may not be the ideal source of information because, as the norm in Western societies is to treat all children equally, they may try to present their investments as being equal across all their grandchildren^9,17^. Parents, in turn, may think of grandparents as couples, meaning they may not accurately report the amount of grandparental investment within lineages. Finally, if one is interested in the investment of all four grandparent types, it would be very complicated to ask either grandparents or parents about the grandparental investment according to all the different grandparent-grandchild dyads. Because of these limitations related to surveying parents and grandparents, children could be the most reliable source of information on biased grandparental investment.

The most important data limitation of this research is that these data did not contain records of potential statistical confounding variables like grandparental resources. Moreover, had those variables been recorded, adolescent children may not be aware of all their grandparents’ backgrounds or even their parents’ background factors important to the questions at hand. By allowing for error covariances (i.e., unexplained aspects of the variation) among latent variables measuring grandparental investment, our estimates should account at least for some unmeasured factors influencing the grandparental investments within lineages^37^. Moreover, we performed within-lineage sensitivity analysis to evaluate the consequences of potential unmeasured confounding on our conclusions. This sensitivity analysis showed that if we consider twice as strong confounding compared to the reported associations by our base model, some of our conclusions changed. Because we have no empirical estimates on how strong effects (for example, parental resource availability) has on the variables studied here, it is hard to argue how big of a problem confounding is to our conclusions. Moreover, it is possible that the level of grandparental investment is related to the probability of being included in the analysis, since the oldest-old grandparents probably invested less and their odds of being alive during the study were lower compared to younger grandparents. This may have particularly pertained to paternal grandfathers, who showed the lowest rate of being alive in these data. This might have produced non-random missingness (MNAR) into the data and analysis^38^. Modelling this selection could have been an option had we had any variables reliably predicting the non-random missingness (e.g. the age of death for the deceased grandparents), which are not part of the main equations of interest^38^. Such selection models also rely on assumptions that are unlikely to be met in real data sets, thus potentially increasing, not decreasing, the bias of the results^38^. Finally, our survey was based on a cross-sectional design, and longitudinal data could be used to obtain a more reliable causal inference on how the death of one grandparent tends to influence the investment of other grandparent types.

The present study investigated whether the survival status of a particular grandparent influenced the investment of other grandparent types within and between lineages. Our findings are based on data from adolescent grandchildren, and future studies are needed to replicate these findings and detect whether the same effect exists if one investigates grandparental investment in younger grandchildren. Moreover, the present study used data from contemporary England and Wales, but the results could be different in other societies, and ideally one should conduct a multinational study. Finally, longitudinal studies including potential confounders are needed to infer causation concerning whether and how other grandparent types will respond to the death of a particular grandparent.

## Supplementary Information

Supplementary Tables.

## Data Availability

The data we used in this study—generated by a third-party—is freely available from https://beta.ukdataservice.ac.uk/datacatalogue/studies/study?id=6075#!/details, but please be aware that, as the data are ‘safeguarded’ (https://www.ukdataservice.ac.uk/get-data/data-access-policy), a user will be required to register for the UK Data Service to be able to access the data. The authors did not had any special access privileges to these data that future researchers would not have.
