# Supplementary material for: The interplay of grandparental investment according to the survival status of other grandparent types
Source: Sci Rep. 2022 Aug 23;12:14390. doi: 10.1038/s41598-022-18693-9 (PMC9399083; doi:10.1038/s41598-022-18693-9)
Supplement: Supplementary file 1 — Supplementary Tables. [file 41598_2022_18693_MOESM1_ESM.pdf]

Electronic supplementary material for "**The interplay of grandparental investment according to the survival status of other grandparent types**"

Samuli Helle, Antti O. Tanskanen, Jenni E. Pettay & Mirkka Danielsbacka

Department of Social Research, University of Turku, Turku, Finland.

Correlations of the variables considered in this study with observed variables used to measure latent constructs "grandparental investment"

Table S1. Correlations among the independent variables included in the analysis and the observed variables used to measure the latent variable "grandparental investment": "how often do you see them" (Q15), "their grandparents had looked after them" (Q26), "they could depend on their grandparents" (Q27) and "provided financial assistance" (Q38). Polychoric correlations are given between binary and ordinal variables and polyserial correlations are given for continuous and ordinal variables. The prefixes MGM, MGF, PGM and PGF denote maternal grandmother, maternal grandfather, paternal grandmother and paternal grandfather, respectively. Correlation coefficients in bold are regarded as statistically significant ($\alpha < 0.05$) after correcting for false discovery rate using the Benjamini-Hochberg method.

	Q15	Q26	Q27	Q38
Maternal grandmothers				
MGF alive	0.107	**0.156**	**0.174**	0.084
PGM alive	0.088	0.091	**0.118**	0.068
PGF alive	0.087	0.074	**0.117**	0.061
Grandchild age	-0.045	**-0.202**	-0.01	-0.041
Maternal grandfathers				
MGM alive	-0.087	0.083	-0.001	5E-04
PGM alive	-0.067	0.009	0.003	-0.043
PGF alive	-0.004	-0.002	0.016	0.005
Grandchild age	-0.006	**-0.184**	-0.003	-0.061
Paternal grandmothers				
MGM alive	-0.105	0.016	-0.051	-0.051
MGF alive	-0.028	0.069	0.073	-0.027

PGF alive	0.045	0.04	0.081	0.028
Grandchild age	**-0.092**	**-0.253**	-0.067	**-0.093**
Paternal grandfathers				
MGF alive	-0.049	0.062	0.043	-0.089
PGM alive	0.036	0.07	0.077	-0.043
PGF alive	0.083	0.144	0.116	0.001
Grandchild age	-0.088	**-0.24**	-0.06	-0.081

Table S2. Biserial correlations among the survival status of grandparents with grandchild's age. The prefixes MGM, MGF, PGM and PGF denote maternal grandmother, maternal grandfather, paternal grandmother and paternal grandfather, respectively. Correlation coefficients in bold are regarded as statistically significant ($\alpha < 0.05$) after correcting for false discovery rate using the Benjamini-Hochberg method.

	Grandchild age
MGM alive	**-0.094**
MGF alive	**-0.073**
PGM alive	-0.051
PGF alive	-0.019

Full results for Bayesian structural equation model

Table S3. Full results for Bayesian structural equation model. The prefixes MGM, MGF, PGM and PGF denote maternal grandmother, maternal grandfather, paternal grandmother and paternal grandfather, respectively. 95% C.I. denotes a 95% credibility interval of the posterior median of coefficients. Grandparental investment: “how often do you see them” (Q15), “their grandparents had looked after them” (Q26), “they could depend on their grandparents” (Q27) and “provided financial assistance” (Q38).

	Median	95% C.I. Lower 2.5%	95% C.I. Upper 2.5%
Structural model			
Investment_MGM			
MGF alive	0.157	0.060	0.256
PGM alive	0.057	-0.047	0.155
PGF alive	0.059	-0.030	0.150
Grandchild age	-0.061	-0.093	-0.030
Investment_MGF			
MGM alive	0.044	-0.199	0.104
PGM alive	-0.033	-0.160	0.088
PGF alive	0.009	-0.101	0.114
Grandchild age	-0.061	-0.099	-0.023
Investment_PGM			
MGM alive	-0.207	-0.349	-0.067
MGF alive	-0.001	-0.117	0.119
PGF alive	0.058	-0.055	0.179
Grandchild age	-0.122	-0.163	-0.082
Relationship_PGF			
MGM alive	-0.207	-0.367	-0.050
MGF alive	-0.025	-0.159	0.108
PGM alive	0.062	-0.123	0.239
Grandchild age	-0.122	-0.169	-0.079
Measurement model			
Factor loadings			
Q38	1.000		
Q26	2.539	2.179	2.928
Q27	1.314	1.188	1.433
Q15_MGM	1.973	1.688	2.269
Q15_MGF	1.658	1.399	1.915
Q15_PGM	1.299	1.109	1.501
Q15_MGF	1.197	1.004	1.389

Thresholds

Q15_1$1	-2.271	-2.537	-1.987
Q15_1$2	0.124	-0.100	0.334
Q15_1$3	1.745	1.503	2.013
Q15_2$1	-2.313	-2.253	-1.977
Q15_2$2	-0.191	-0.460	0.074
Q15_2$3	1.421	1.137	1.707
Q15_3$1	-2.208	-2.487	-1.921
Q15_3$2	0.111	-0.101	0.325
Q15_3$3	1.709	1.473	1.961
Q15_4$1	- 1.838	-2.148	-1.557
Q15_4$2	0.219	-0.029	0.474
Q15_4$3	1.885	1.598	2.183
Q26_1$1	-0.530	-0.807	-0.253
Q26_1$2	1.483	1.193	1.779
Q26_1$3	3.777	3.347	4.221
Q26_2$1	-0.855	-1.288	-0.465
Q26_2$2	1.150	0.748	1.566
Q26_2$3	3.784	3.256	4.378
Q26_3$1	-0.661	-1.095	-0.265
Q26_3$2	1.584	1.172	2.012
Q26_3$3	4.567	3.924	5.192
Q26_4$1	-0.411	-0.929	0.112
Q26_4$2	1.783	1.272	2.295
Q26_4$3	4.843	4.136	5.603
Q27_1$1	-1.428	-1.599	-1.250
Q27_1$2	-0.627	-0.719	-0.479
Q27_1$3	0.185	0.032	0.342
Q27_2$1	-1.544	-1.769	-1.308
Q27_2$2	-0.732	-0.949	-0.520
Q27_2$3	0.069	-0.133	0.292
Q27_3$1	-1.517	-1.750	-1.282
Q27_3$2	-0.658	-0.880	0.437
Q27_3$3	0.248	0.026	0.458
Q27_4$1	-1.358	-1.626	-1.059
Q27_4$2	-0.566	-0.836	-0.286
Q27_4$3	0.371	0.090	0.638
Q38_1$1	-1.402	-1.546	-1.245
Q38_1$2	0.045	-0.081	0.171
Q38_2$1	-1.361	-1.557	-1.179
Q38_2$2	-0.086	-0.259	0.084
Q38_3$1	-1.483	-1.678	-1.293
Q38_3$2	-0.052	-0.225	0.125
Q38_4$1	-1.298	-1.548	-1.074
Q38_4$2	0.060	-0.158	0.280

Error variances

Investment_MGM	0.452	0.365	0.543
Investment_MGF	0.599	0.481	0.730
Investment_PGM	0.656	0.529	0.791
Investment_PGF	0.747	0.588	0.905
Error covariances of latent variables			
Investment_MGM with			
Investment_MGF	0.462	0.384	0.553
Investment_PGM	0.133	0.086	0.181
Investment_PGF	0.118	0.068	0.173
Investment_MGF with			
Investment_PGM	0.181	0.125	0.240
Investment_PGF	0.166	0.107	0.230
Involvement_PGM with			
Investment_PGF	0.669	0.551	0.799
